# Editorial: Regulatory Mechanisms of Leaf Senescence Under Environmental Stresses

**DOI:** 10.3389/fpls.2020.01293

**Published:** 2020-08-19

**Authors:** Yasuhito Sakuraba, Jinjie Li, Soyon Park, Nam-Chon Paek

**Affiliations:** ^1^ Biotechnology Research Center, The University of Tokyo, Tokyo, Japan; ^2^ Key Lab of Crop Heterosis and Utilization of Ministry of Education, China Agricultural University, Beijing, China; ^3^ Division of Plant Science, University of Missouri, Columbia, MO, United States; ^4^ Department of Plant Science, Seoul National University, Seoul, South Korea

**Keywords:** leaf senescence, stay green, environmental stress, phytohormones, transcription factors

Leaf senescence defines the final stage of the leaf developmental program and is characterized by extensive destabilization of intracellular organelles and decomposition of macromolecules in order to relocate nutrients to the actively developing organs. Over the last two decades, a number of genes associated with leaf senescence have been identified, thus significantly enhancing our understanding of the regulatory mechanisms underlying this phenomenon (Woo et al., 2019; Sakuraba et al., 2020). However, given the complexity of leaf senescence, many molecular mechanisms and associated factors involved in this process might still be unknown.

The purpose of this Research Topic is to identify the genes or regulatory mechanisms associated with environmental stress-induced leaf senescence or the regulatory mechanisms underlying environmental stress responses that are potentially important for the regulation of leaf senescence. In this Research Topic, a total of 10 research articles have been accepted for publication; these articles focus on a wide range of plant species, including the model plant *Arabidopsis thaliana* and various crops such as rice (*Oryza sativa*), tobacco (*Nicotiana tabacum*), and cotton (*Gossypium hirsutum*).

Light deprivation is one of the many environmental stresses that induce leaf senescence. In *Arabidopsis*, dark-induced leaf senescence requires phytochrome-interacting transcription factors (TFs), PIF4 and PIF5 (Sakuraba et al., 2014), as well as phytohormones such as abscisic acid (ABA) and ethylene. In this Research Topic, Ueda et al. investigated the relationship between ethylene, ABA, and PIFs in dark-induced leaf senescence in *Arabidopsis*. The *pif4 pif5* double mutant exhibited delayed yellowing during dark-induced leaf senescence. However, they showed that during ABA-induced leaf senescence under light, the *pif4 pif5* double mutant did not show decreased sensitivity to ABA, suggesting that PIF4 and PIF5 act upstream of ABA signaling. On the other hand, the triple mutant of *pif4 pif5* and ethylene-insensitive *ein2* exhibited a stronger delayed senescence phenotype than the *ein2* single mutant and *pif4 pif5* double mutant, suggesting that EIN2-mediated ethylene signaling and PIF4/PIF5 independently regulate dark-induced leaf senescence.

NAC and WRKY TFs are considered to play important roles in the regulation of leaf senescence. In this Research Topic, Doll et al. reported that *Arabidopsis* WRKY25 acts as a negative regulator of hydrogen peroxide (H_2_O_2_)-mediated promotion of leaf senescence. WRKY25 binds to and enhances the activity of the promoter of the *WRKY53* gene, which encodes a key TF that promotes leaf senescence; however, WRKY25 directly represses the activity of its own gene promoter. Additionally, Doll et al. showed that MEKK1, a component of the mitogen-activated protein kinase (MAPK) signaling pathway, enhances the ability of WRKY25 to activate the *WRKY53* promoter. Thus, WRKY25 and WRKY53 form a highly complex and robust regulatory network to regulate H_2_O_2_-mediated promotion of leaf senescence. On the other hand, Gu et al. reported that in cotton (*Gossypium hirsutum*), GhWRKY91 acts as a negative regulator of leaf senescence. Constitutive expression of *GhWRKY91* in *Arabidopsis* delayed leaf yellowing during natural leaf senescence and under dehydration stress. Furthermore, they demonstrated that GhWRKY91 activates the promoter of *GhWRKY17*, which is involved in ABA signaling and reactive oxygen species (ROS) production.

Compared with NAC and WRKY TFs, roles of other TFs in the regulation of leaf senescence remain unclear. Zhang et al. reported that DEAR4, a DREB/CBF family TF, acts as a positive regulator of leaf senescence in *Arabidopsis*. Transgenic *Arabidopsis* plants overexpressing *DEAR4* exhibited accelerated leaf yellowing during natural and dark-induced senescence. In addition, they also showed that *DEAR4* overexpressing plants were more sensitive to high salinity and drought stresses than wild-type plants, and DEAR4 increased the sensitivity to these environmental stresses probably by enhancing ROS production. On the other hand, Lim et al. identified a novel senescence-associated AP2/ERF family TF in rice, ETHYLENE RESPONSE FACTOR 101 (OsERF101). OsERF101 directly activates the transcription of genes encoding OsNAP and OsMYC2 TFs, both of which activate genes associated with chlorophyll degradation and jasmonate (JA) signaling.

A balance between carbon (C) and nitrogen (N) availability is one of the key determinants affecting the progression of leaf senescence. *Arabidopsis* plants grown under N-deficient conditions with elevated CO_2_ levels exhibited precocious leaf senescence (Aoyama et al., 2014). Li et al. identified several key components of the C/N-nutrient response including a leucine-rich repeat receptor-like kinase with extracellular malectin-like domain (LMK1) using phosphoproteomics approaches. Further analyses revealed that LMK1 exhibits cell death induction activity in plant leaves. Thus, LMK1 potentially acts as a key regulator of the progression of C/N imbalance-induced leaf senescence.

Cytokinin (CK) negatively regulates leaf senescence and enhances tolerance to environmental stresses, such as drought and high salinity. In tobacco, constitutive expression of *ISOPENTENYL TRANSFERASE* (*IPT*), which encodes a key CK biosynthetic enzyme, delayed leaf senescence but also caused negative growth phenotypes, such as dwarfism and root growth inhibition (Smart et al., 1991). In this Research Topic, Avni et al. reported that transgenic tobacco plants overexpressing the *IPT* gene under the control of the stress-inducible promoter of the *Arabidopsis* *METALLOTHIONEIN* gene were tolerant to dehydration and high salinity stresses and showed normal growth and metabolic maintenance. Thus, environmental stress-specific induction of CK biosynthesis is a useful approach for developing plants with improved biomass and yield under environmental stress conditions, and this approach can be applied to various crop species.

The phytohormone ABA is closely associated with the drought stress response. In this Research Topic, three studies identified proteins involved in the ABA-dependent drought stress response in *Arabidopsis*. Baek et al. reported the role of *Arabidopsis* PR5 receptor-like kinase 2 (AtPR5K2) in the drought stress response. They showed that AtPR5K2 physically interacts with and phosphorylates Type 2C protein phosphatases, ABA-INSENSITIVE 1 (ABI1) and ABI2, which regulate the initiation of ABA signaling. Baek et al. investigated the role of *Arabidopsis* RPD3-type HISTONE DEACETYLASE 9 (HDA9) in the ABA-dependent drought stress response. HDA9 physically interacts with ABA-INSENSITIVE 4 (ABI4), a key ABA signaling TF, and the results of chromatin immunoprecipitation and quantitative PCR (ChIP-qPCR) indicated that the HDA9–ABI4 complex directly represses the expression of *CYP707A1* and *CYP707A2* genes, which encode ABA catabolic enzymes. On the other hand, a previous study showed that the HDA9–POWERDRESS (PWR) complex participates in the regulation of leaf senescence, flowering time, and floral dormancy. Khan et al. showed that both HDA9 and PWR interact with ABI4, probably forming the PWR–HDA9–ABI4 complex, and repress the expression of genes associated with ABA metabolism and signaling.

In conclusion, we believe that studies included within the Research Topic “Regulatory Mechanisms of Leaf Senescence Under Environmental Stress” improve our understanding of the molecular mechanisms of environmental stress-induced leaf senescence. We sincerely appreciate all scientists who kindly allowed us to publish their work in this Research Topic.

## Author Contributions

All authors listed have made substantial, direct, and intellectual contribution to the work and approved it for publication.

## Funding

YS is supported by a grant from JSPS KAKENHI (grant no. 17H05024).

## Conflict of Interest

The authors declare that the research was conducted in the absence of any commercial or financial relationship that could be constructed as a potential conflict of interest.
